# Bone regeneration of a polymeric sponge technique—Alloplastic bone substitute materials compared with a commercial synthetic bone material (MBCP+TM technology): A histomorphometric study in porcine skull

**DOI:** 10.1002/cre2.394

**Published:** 2021-01-06

**Authors:** Punyada Intapibool, Naruporn Monmaturapoj, Katanchalee Nampuksa, Kriangkrai Thongkorn, Pathawee Khongkhunthian

**Affiliations:** ^1^ Faculty of Dentistry, Center of Excellence for Dental Implantology Chiang Mai University Chiang Mai Thailand; ^2^ National Science and Technology Development Agency Bangkok Thailand; ^3^ Faculty of Veterinary Medicine, Department of Companion Animal and Wildlife Clinic Chiang Mai University Chiang Mai Thailand

**Keywords:** biphasic calcium phosphate, bone substitute materials, histomorphometry, pig, porosity

## Abstract

**Background:**

Polymeric sponge technique is recommended for developing the desired porosity of Biphasic calcium phosphate (BCP) which may favor bone regeneration.

**Purpose:**

To investigate the healing of BCP with ratio of HA30/β‐TCP70 (HA30) and HA70/β‐TCP30 (HA70) polymeric sponge preparation, compare to commercial BCP (MBCP+TM).

**Materials and Methods:**

Materials were tested X‐ray diffraction (XRD) pattern and scanning electron microscope (SEM) analysis. In eight male pigs, six calvarial defects were created in each subject. The defects were the filled with 1 cc of autogenous bone, MBCP+TM (MBCP), HA30, HA70, and left empty (negative group). The new bone formations, residual material particles and bone‐to‐graft contacts were analyzed at 4, 8, 12 and 16 weeks.

**Results:**

Fabricated BCP showed well‐distributed porosity. At 16 weeks, new bone formations were 45.26% (autogenous), 33.52% (MBCP), 24.34% (HA30), 19.43% (HA70) and 3.37% (negative). Residual material particles were 1.88% (autogenous), 17.58% (MBCP), 26.74% (HA30) and 37.03% (HA70). These values were not significant differences (Bonferroni correction <0.005). Bone‐to‐graft contacts were 73.68% (MBCP), which was significantly higher than 41.68% (HA30) and 14.32% (HA70; Bonferroni correction <0.017).

**Conclusions:**

Polymeric sponge technique offers well‐distributed porosity. The new bone formation and residual material particles were comparable to MBCP+TM, but the bone‐to‐graft contact was lower than MBCP+TM.

## 1 INTRODUCTION

On account of bone remodeling in dental sockets, the alveolar crests result in progressive changes of their dimensions (Araujo et al., 2015; Chappuis et al., 2017) and qualities (Kuroshima et al., 2017). Consequently, insufficient ridge amounts can occur which challenges dental implant treatment in both esthetics and functional aspects. Bone augmentation is one of the procedures that can resolve this problem (Roden Jr., 2010). Autogenous bone remains considered as the gold standard graft because it consists of all bone formation characteristics which are osteogenesis, osteoinduction and osteoconduction. However, there are some concerns about the surgery at the donor sites that may cause patient morbidity. Moreover, only a limited amount of the graft can be taken and their resorption rate is unpredictable (Marx, 2007; Precheur, 2007; Roden Jr., 2010; Sheikh et al., 2017; Thrivikraman et al., 2017). Other grafts have been developed to fulfill these autograft's disadvantages but these grafts contain just osteoconduction and possible osteoinduction. Allograft and xenograft are common bone substitutions (Marx, 2007; Precheur, 2007; Roden Jr., 2010; Sheikh et al., 2017; Thrivikraman et al., 2017). By contrast, they could cause disease transmission (Boneva et al., 2001; Mirabet et al., 2017). Therefore, alloplastic grafts has been fabricated (Sheikh et al., 2017). The systematic reviews show no significant different outcomes among the bone substitutions in bone augmentation procedures (Cecilia et al., 2018; Corbella et al., 2016; Nawas & Schiegnitz, 2014) and alveolar ridge preservation (De Risi et al., 2015). In addition, the alloplast results in comparable percentage of new bone formation (Papageorgiou et al., 2016; Wu et al., 2016) and implant treatment outcomes in maxillary sinus floor augmentation (Starch‐Jensen et al., 2018) to other materials. However, the alloplast yields lower bone‐to‐graft contact than xenograft (Wu et al., 2016).

One of the most popular alloplasts is biphasic calcium phosphate (BCP) because its chemical structure has a close resemblance to the inorganic part of the human bone. Furthermore, their biocompatibility and bioactivity are mandatory for bone regeneration (Dorozhkin, 2007, 2010, 2013, 2016). BCP composes of Hydroxyapatite (HA) and β‐tricalcium phosphate (β‐TCP). HA has excellent biocompatibility and more stable phase which facilitate the space‐maintaining capacity (Kattimani et al., 2016; Moller et al., 2014). β‐TCP shows more soluble phase and resorbs into calcium and phosphate ions. These ions precipitate the bone‐making cells to the regenerated site which will promote new bone formation (Asvanund & Chunhabundit, 2012; Bettach et al., 2014; Piccinini et al., 2016). These two compositions benefit in the balance between the new bone formation and the bone graft resorption rate (Dorozhkin, 2007, 2010, 2013, 2016). As a result, the HA/β‐TCP ratio effects the bone regeneration process. Higher HA/β‐TCP ratio exhibits good osteoconductive properties (Ebrahimi et al., 2014; Pripatnanont et al., 2016) while lower HA/β‐TCP ratio shows more new bone formation area (Jelusic et al., 2017; Jensen et al., 2007; Wang et al., 2013; Yang et al., 2014; Zhang et al., 2005; Zhu et al., 2010). There are a number of studies which demonstrate BCP's properties. In in vitro studies, BCP displays biocompatibility (Bakhtiari et al., 2010; Bernhardt et al., 2011; Piccinini et al., 2016) which can promote the proliferation of osteoblasts (Bernhardt et al., 2013; Lomelino Rde et al., 2012) and mesenchymal stem cells (Lobo et al., 2015). In animal studies, BCP shows positive potential in bone regeneration capacities (Alsayed et al., 2015; Asvanund & Chunhabundit, 2012; Choi et al., 2011; Dahlin et al., 2015; Ezirganli et al., 2015; Khan et al., 2015; Lindhe et al., 2013; Macedo et al., 2014; Macedo et al., 2018; Mendoza‐Azpur et al., 2015; Park et al., 2010; Piccinini et al., 2013; Suaid et al., 2013, 2014; Sungtae et al., 2011; Yazdi et al., 2013; Yip et al., 2015). In clinical studies BCP exhibits good results in terms of new bone formation and volume stability in maxillary sinus augmentations (Bettach et al., 2014; Chiu et al., 2018; Christer et al., 2009, 2012; Covani et al., 2011; Danesh‐Sani et al., 2016; Frenken et al., 2010; Helder et al., 2018; Iezzi et al., 2012; Lee et al., 2018; Mangano et al., 2013; Nishimura et al., 2018; Ohe et al., 2016; Okada et al., 2017; Schmitt et al., 2013; Sebastian et al., 2010; Sverzut et al., 2015; Tosta et al., 2013) and contouring of alveolar ridges after immediate implant placement (Assaf et al., 2013).

BCP is considered as an osteoconductive scaffold. Grain size, pore size and porosity play important roles for the scaffold property. Small grain size (<1 mm) scaffolds are preferably resorbed by osteoclast‐like cells and have better shapeability while large grain size allows superior space‐making capability (Dorozhkin, 2010; Yamada & Egusa, 2017). Apart from the grain size, pore size is crucial for cell migration and tissue ingrowth. Pore size can be categorized in macroporous, microporous and interconnectivity. Macroporous (>100 μm) is associated with the migration and proliferation of osteoblasts and mesenchymal cells as well as the mineralized bone ingrowth. Microporous (<10 μm) enhances protein adsorption and body fluid exchange. Likewise, interconnectivity assists the vascularity which includes nutrient contribution and waste removal (Hannink & Arts, 2011; Klein et al., 2009; Mate Sanchez de Val, Calvo‐Guirado, Gomez‐Moreno, Gehrke, et al., 2016; Mate Sanchez de Val, Calvo‐Guirado, Gomez‐Moreno, Perez‐Albacete Martinez, et al., 2016). Higher porosities result in larger surface area which encourage cell adhesion and bone ingrowth but this condition will reduce the mechanical resistance of bone graft (Calvo‐Guirado et al., 2015; Mate Sanchez de Val et al., 2015; Yamada & Egusa, 2017). One study reported that the total porosities should be 70% of the bioceramic volume (Dorozhkin, 2010).

To control the pore size and porosity is a complicated engineering fabrication (Bose et al., 2012; Dorozhkin, 2007). One of the recommended manufacturing methods is the polymeric sponge technique (Aghajani et al., 2017; Naqshbandi et al., 2013; Wang et al., 2017). In this technique, the material is prepared by milling the slurry BCP on the controlled porosity polyurethane template (Ebrahimi et al., 2012; Ebrahimi et al., 2014). Moreover, the property of the graft material is also influenced by the manufacturing process. High sintering temperature enhances the graft's crystallinity which results in high bone graft density and crystal size (Mate Sanchez de Val, Calvo‐Guirado, Gomez‐Moreno, Perez‐Albacete Martinez, et al., 2016; Yamada & Egusa, 2017). This condition impairs the degradation of the materials (Araujo et al., 2010).

However, there has not been any study regarding bone regeneration potential of BCP fabricated by the polymeric sponge method. Therefore, the grafts were to be evaluated in augmentation procedures in experimentally created critical size defects of the adult pigs by histomorphometry.

## 2 MATERIALS AND METHODS

### 2.1 Material characterization

The alloplastic materials were fabricated by polymeric sponge method in HA:β‐TCP 30:70 (HA30) and 70:30 (HA70) volumetric compositions from the same batches of materials by National Science and Technology Development Agency (NSTDA), Thailand. The sintering temperature was controlled at 600°c followed by increasing to 1000°c with 2 h per each control. In order to identify the phase's composition, the materials were tested by X‐ray diffraction (XRD). XRD patterns were obtained on the two ratios of BCP particles in an X‐ray diffractometer (PANalytical) using Cu‐Ka radiation (40 mA, 30 kV). Scans were achieved with 2θ values from 20° to 60° at a rate of 0.02°/min.

The fabricated materials were compared with MBCP+TM (Biomantlante, France). MBCP+TM is the commercial BCP which composes of HA:β‐TCP 20:80 by volume. A scanning electron microscope (JEOL, Japan) was used to study the morphology of all the test particles. The samples were pre‐coated using a carbon evaporation coating unit. They were examined using SEM at a working distance of 10 mm, at an acceleration voltage of 5 kV.

### 2.2 Surgical procedure

The animal experimental study was designed. The Institutional Animal Care and Use Committee, Faculty of Medicine, Chiang Mai University approved the animal experimental study protocol (No. 29/2560). Eight 50–60 kg (4–5 months) domestic male pigs (*Sus scrofa domesticus*) were acclimatized 1 week then randomized into four groups in order to investigate bone healing at 4 weeks (*n* = 2), 8 weeks (*n* = 2), 12 weeks (*n* = 2) and 16 weeks (*n* = 2). NPO (with holding oral intake of food and fluids) was decided in each pig at least 12 h before an intramuscular injection of zoletil 5 mg/kg bodyweight and xylazine 2 mg/kg bodyweight premedication. Atropine 0.05 mg/kg bodyweight and midazolam 0.5 mg/kg bodyweight were injected intravenously through an ear vein for the induction. The animal was given an endotracheal intubation and a controlled respiration frequency of 12 per minute with a volume of 10 ml/kg body weight. Isoflurane 1.0%–1.5% was maintained with a mixture of oxygen.

**FIGURE 1:**
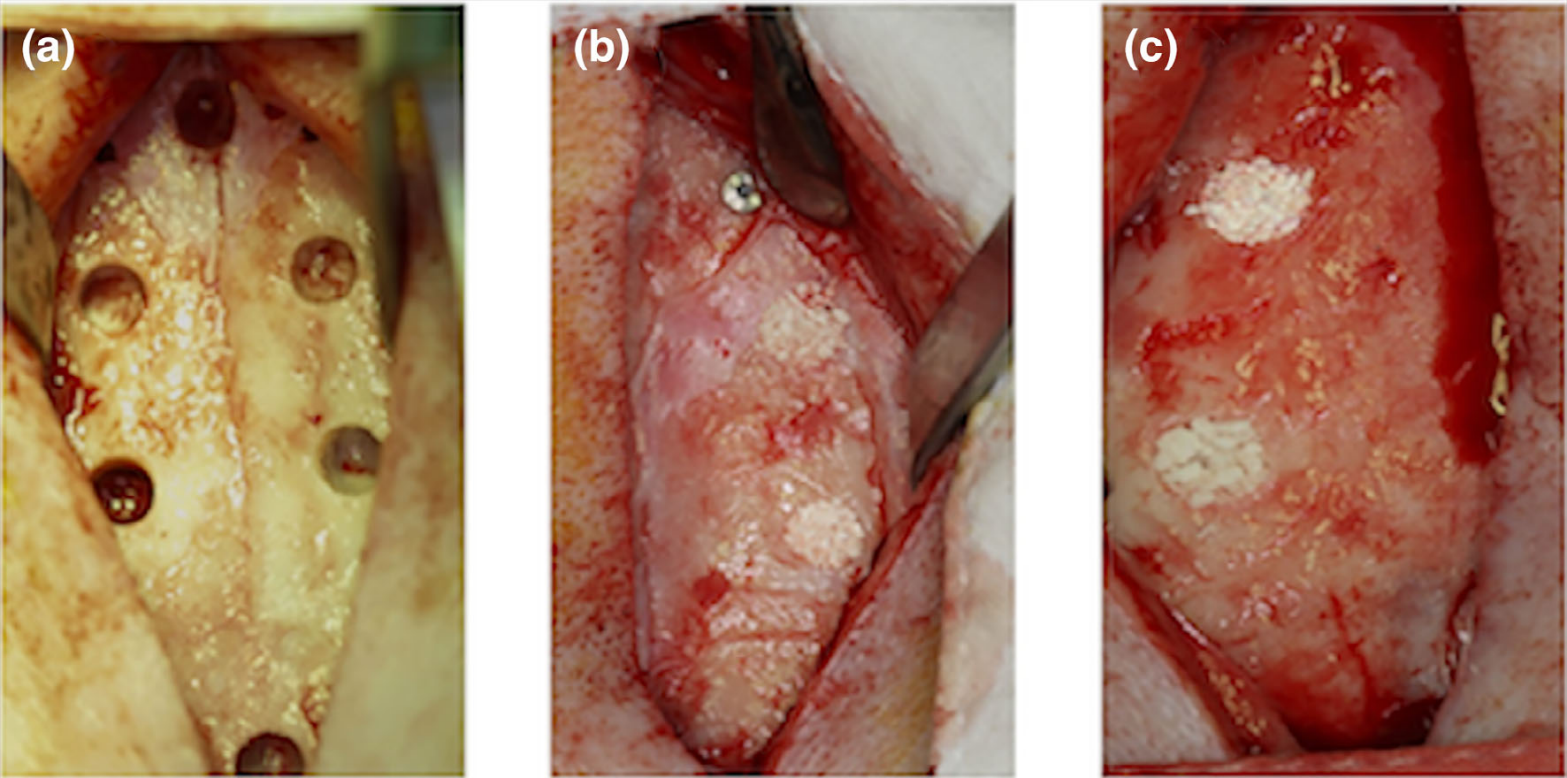
The surgical procedure. (a) Six calvarial defects were created. (b) Each of 1 cc autogenous, fabricated BCP with HA70 and MBCP+TM were filled. (c), each of 1 cc fabricated BCP with HA30 and MBCP+TM were filled, while the last defect was left empty

The administration of 10 mg/kg of amoxicillin were given intramuscularly for prophylaxis. The calvarial site was scrubbed with 10% povidone iodine. After local infiltration of 4% articaine with 1:100,000 epinephrine 1.7 ml, a midsagittal incision was created. A subperiosteal dissection and six standardized intraosseous defects using a trephine with copious saline irrigation was performed. Each defect dimension was 5.0 mm in diameter and 1.0 mm in depth adapted from ISO 10993‐6: 2016 protocol (ISO 10993‐6, 2016). One defect was filled with 1 cc autogenous bone graft which was milled from the calvarial bone, another two defects were filled with 1 cc MBCP+TM alloplastic graft, 1 cc of the fabricated BCP with HA30 was filled for one defect, whereas 1 cc of the fabricated BCP with HA70 was filled for another defect and the last one was left empty (Figure 1).

The eight animals were divided into four groups each containing two animals and were followed up every 2 weeks. However, each group had a different designated healing period of 4, 8, 12 and 16 weeks respectively. At the end of each designated healing period, the animals were sacrificed by barbiturates overdose intravenous injection.

The calvarium were harvested using a small, sharp fissure bur and then fixed in 10% neutral‐buffered formalin for at least 2 weeks. Each block was dehydrated using a graded series of alcohol and embedded in methyl‐methacrylate for 50 days. Using a low speed diamond saw with copious cooling, a defect was sectioned in a bucco‐lingual direction (Donath & Breuner, 1982). The sections were mounted on opaque Plexiglas with acrylic glue and ground to a final thickness of ~8–10 μm. Finally, the sections were superficially stained with toluidine blue and basic fuchsin (Buser et al., 2004). Each defect was sectioned into two sample slides.

### 2.3 Histologic and histomorphometric analysis

To analyze the histology, all sections were illustrated by light microscope (Axio Lab.A1, Zeiss) with the objective lens set at 5×, 10× and 40×. A digital camera (1200D, Cannon) which was connected to the light microscope was used to capture the images and then stored in a computer. Each specimen was investigated for the quality of new bone formation, residual materials, and inflammatory responses. In addition, the compact stereo microscope (Stemi 305, Zeiss) with the objective lens set at 10× was used to analyze the histomorphometry. The samples were measured with Image J 1.52a (Wayne Rasband National Institute of Health) and Axio Vision SE64 Rel. 4.9.1 (Carl Zeiss Microscopy, LLC).

The histomorphometric values were analyzed by two examiners. The quantity of new bone formation was calculated by the percentage of the newly formed bone area to the total defect area.

$$\left(\%\right)\;\mathrm{new}\;\text{bone formation}=\frac{\text{newly formed bone area}\;\left({\text{pixel}}^{2}\right)}{\text{total defect area}\;\left({\text{pixel}}^{2}\right)}\;x\;100$$

The amount of residual material particles was calculated by the percentage of the grafting material particle area to the total defect area.

$$\left(\%\right)\;\text{residual material particles}=\frac{\text{grafting material particle area}\;\left({\text{pixel}}^{2}\right)}{\text{total defect area}\;\left({\text{pixel}}^{2}\right)}\;x\;100$$

Bone‐to‐graft contact of the biomaterial was calculated by the percentage of the length of bone contact to graft surface to the circumference of the graft particle.

$$\left(\%\right)\;\text{Bone}-\text{to}-\text{graft contact}=\frac{\text{length of bone contact to graft surface}\;\left(\text{pixel}\right)}{\text{graft circumference}\;\left(\text{pixel}\right)}\;x\;100$$

### 2.4 Data analysis

Statistical analysis was performed using SPSS software (SPSS version 15, SPSS Inc.). Due to the limited sample sizes (total animal *n* = 8), non‐parametric tests were used. The data were presented as median (interquartile range). The reliability among two examiners were compared by using intraclass correlation coefficient. To compare the new bone formation, the residual material particles and the bone to graft contact at each healing period (comparing at 4‐week, 8‐week, 12‐week, and 16‐week healing), the differences among groups of the percentage of new bone formation, the residual material particles and the percentage of bone‐to‐graft contact were compared by using both Kruskal–Wallis and Friedman test. After that, the differences of those parameters between the materials of each time frame were analyzed using Mann–Whitney test.

### 2.5 Ethics statement

Certificate of approval for use of animals, Faculty of Medicine, Chiang Mai University. Protocol number: 29/2560.

## 3 RESULTS

**FIGURE 2:**
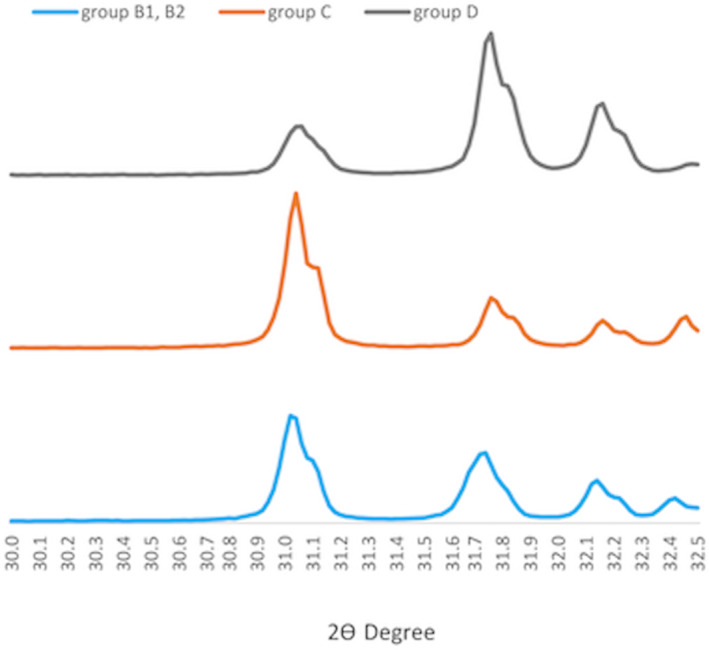
XRD pattern in MBCP group, HA30 group and HA70 group

The phases of the materials were presented by the XRD pattern. The increase in peak height and decrease in peak width indicated that the materials exhibit high crystallinity. Two phases can be identified with no new formed phase in two compositions of the materials. The fabricated BCP showed higher peak height and lower peak width (Figure 2).

**FIGURE 3:**
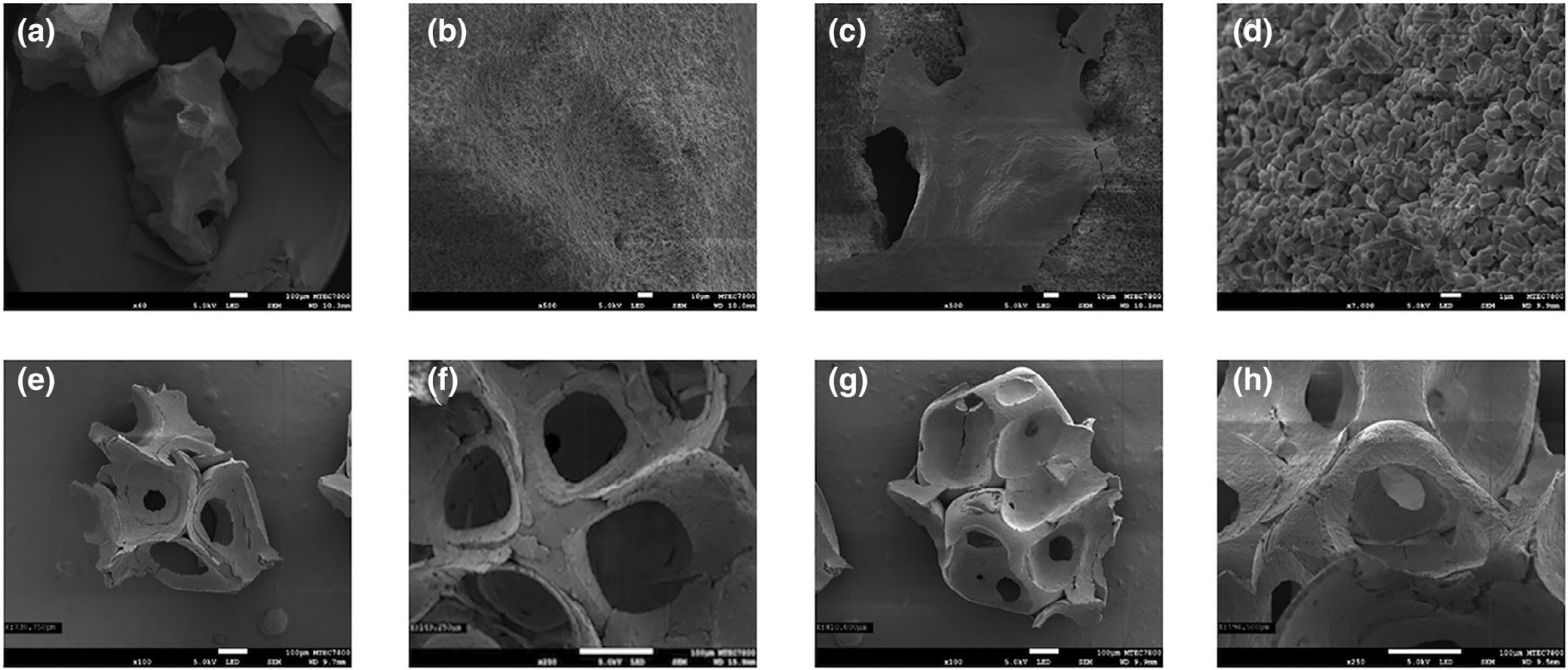
SEM analysis in different biomaterials and magnifications: (MBCP group). (a) ×60 magnification. (b) ×500 magnification. (c) ×500 magnification. (d) ×7000 magnification, (HA30 group). (e) ×100 magnification. (f) ×250 magnification and (HA70 group). (g) ×100 magnification. (h) ×250 magnification

The morphologic analysis of the materials was described using the SEM images. The sizes of fabricated BCP in both compositions range from 1000 to 2000 μm. Irregular and rough surface were detected. Its homogenous structures represent highly dense crystallinity. In addition, well distributed pores were found within the materials. The porosity of the fabricated BCP was over 80%, with pore sizes composed of 100–500 μm (macroporosity) and 1–10 μm (microporosity; Figure 3e,g). Moreover, the interconnected pores were noticed with high magnification (Figure 3f,h). While MBCP+TM's granule sizes range from 500 to 1000 μm (Figure 3a). From the view of macrostructure, the surface appeared flatter. With ×500 magnification view, the tiny porosities and the roughness were observed (Figure 3b). BCP's structure composes of two layers which are the hexagonal plate‐like shape form surrounded with the rod shape forms (Figure 3c). These forms demonstrate small‐scale crystallinity. In addition, with ×7000 magnification, the nano‐scale porosity arises from the boundary from the droplets of the materials (Figure 3d).

Non‐parametric statistics were used in this study, due to the limited number of animals used. The study was strictly concerned about the animal life and followed the principles of the 3Rs (replacement, refinement, reduction). Our study design was minimized the number of animals used and was appropriately analyzed to ensure robust and reproducible findings.

**FIGURE 4:**
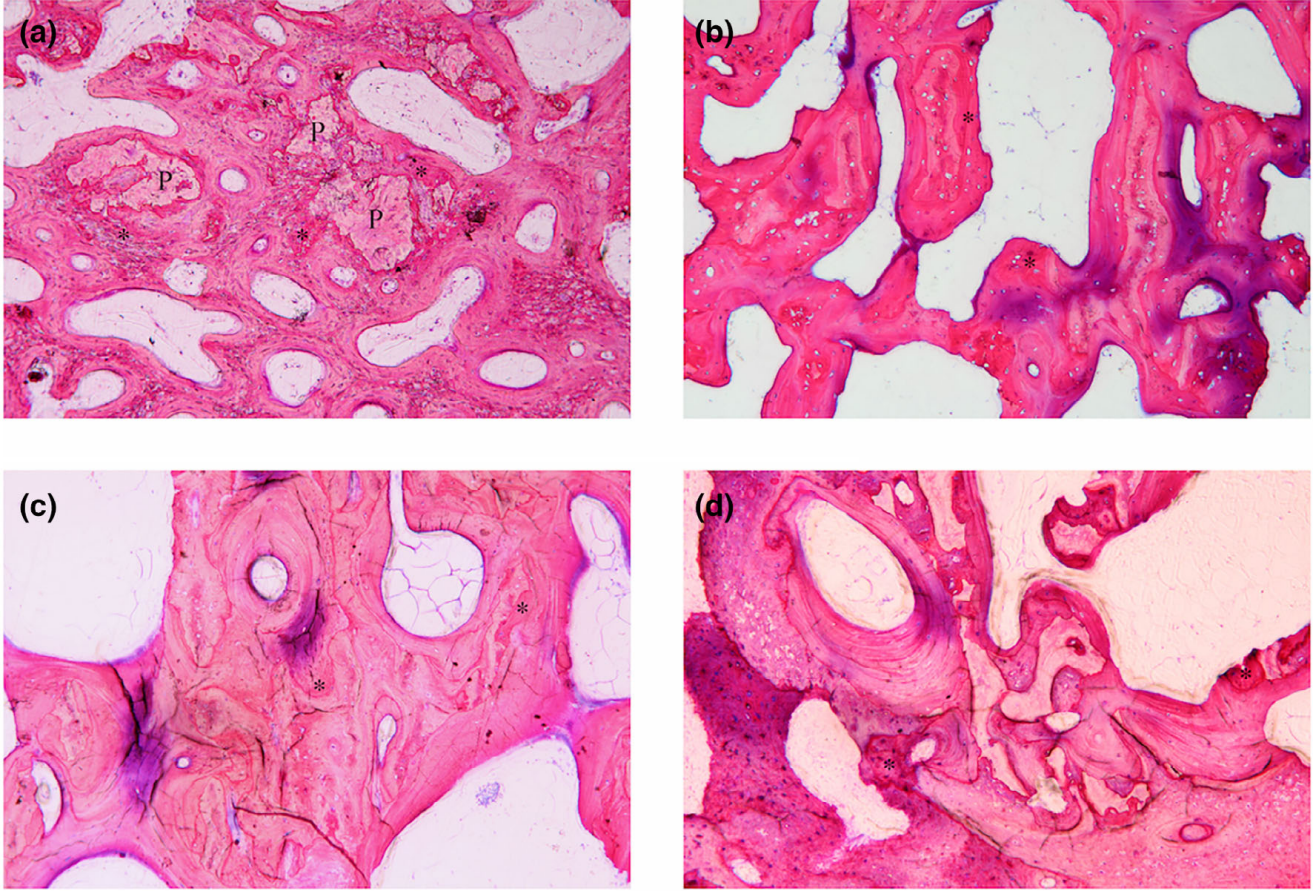
Histologic images of autogenous samples show new bone formation (asterisks) and material remnants (P) in (a) 4 weeks. (b) 8 weeks. (c) 12 weeks. (d) 16 weeks with ×10 magnification

The histology findings were investigated. Two slides of the autogenous samples, one from 8 weeks and the other from 12 weeks were excluded due to poor quality image for analysis. In the autogenous group, the defects showed new osteoid formation which was completely integrated around the remnant grafts in 4 weeks (Figure 4a). The autogenous particles were mainly resorbed and difficult to distinguish from the surrounding bone in 8 weeks. The laminated bone formation was observed in the defects (Figure 4b). In 12 and 16th weeks, the Harvesian's canals were seen (Figure 4c,d).

**FIGURE 5:**
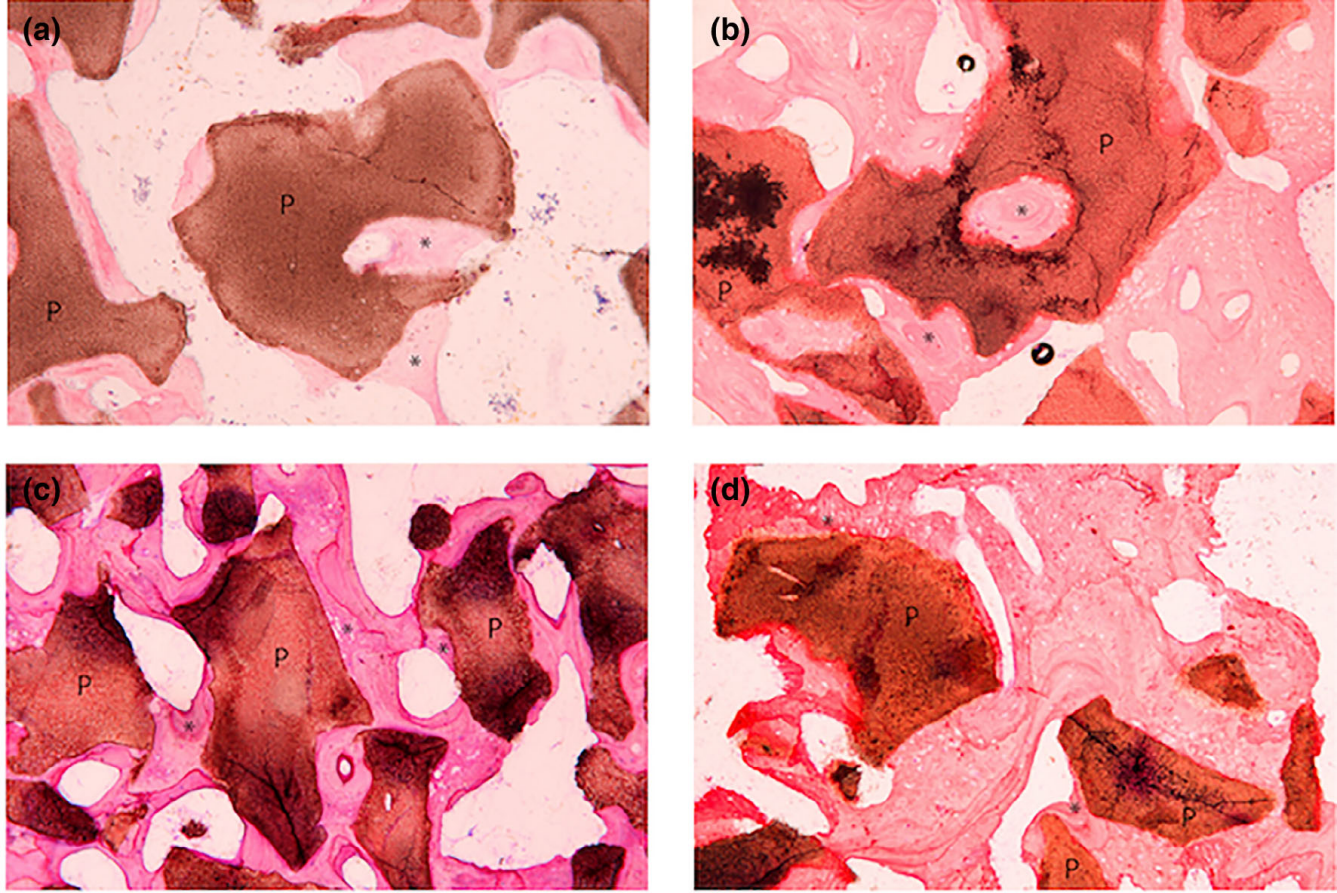
Histologic images of MBCP group samples show new bone formation (asterisks) and material remnants (P) in (a) 4 weeks. (b) 8 weeks. (c) 12 weeks. (d) 16 weeks with ×10 magnification

In group MBCP, the particles showed good distribution. During the first 4 weeks the particles were partially contacted with new woven bone regeneration. The bone formation also occurred within the porous (Figure 5a). In 8 weeks, the lamellar bone was regenerated with more contact bone around the grafts (Figure 5b). The maturation and consolidation of the bone developed over time while the particle sizes were trend to be smaller as seen in 12 and 16 weeks (Figure 5c,d).

**FIGURE 6:**
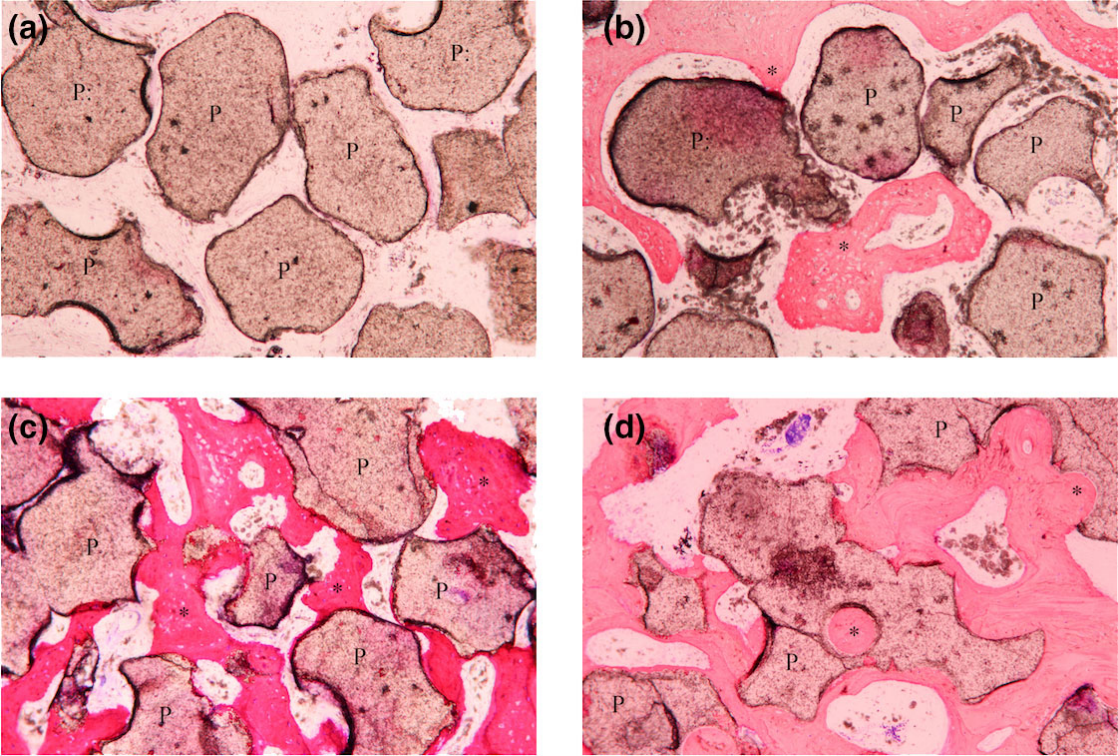
Histologic images show new bone formation (asterisks) and material remnants (P) of (HA30 group) in (a) 4 weeks. (b) 8 weeks. (c) 12 weeks. (d) 16 weeks and (HA70 group) in (e) 4 weeks. (f) 16 weeks: Aggregated particle. (g) 16 weeks: Well‐distributed particle with ×10 magnification

**FIGURE 7:**
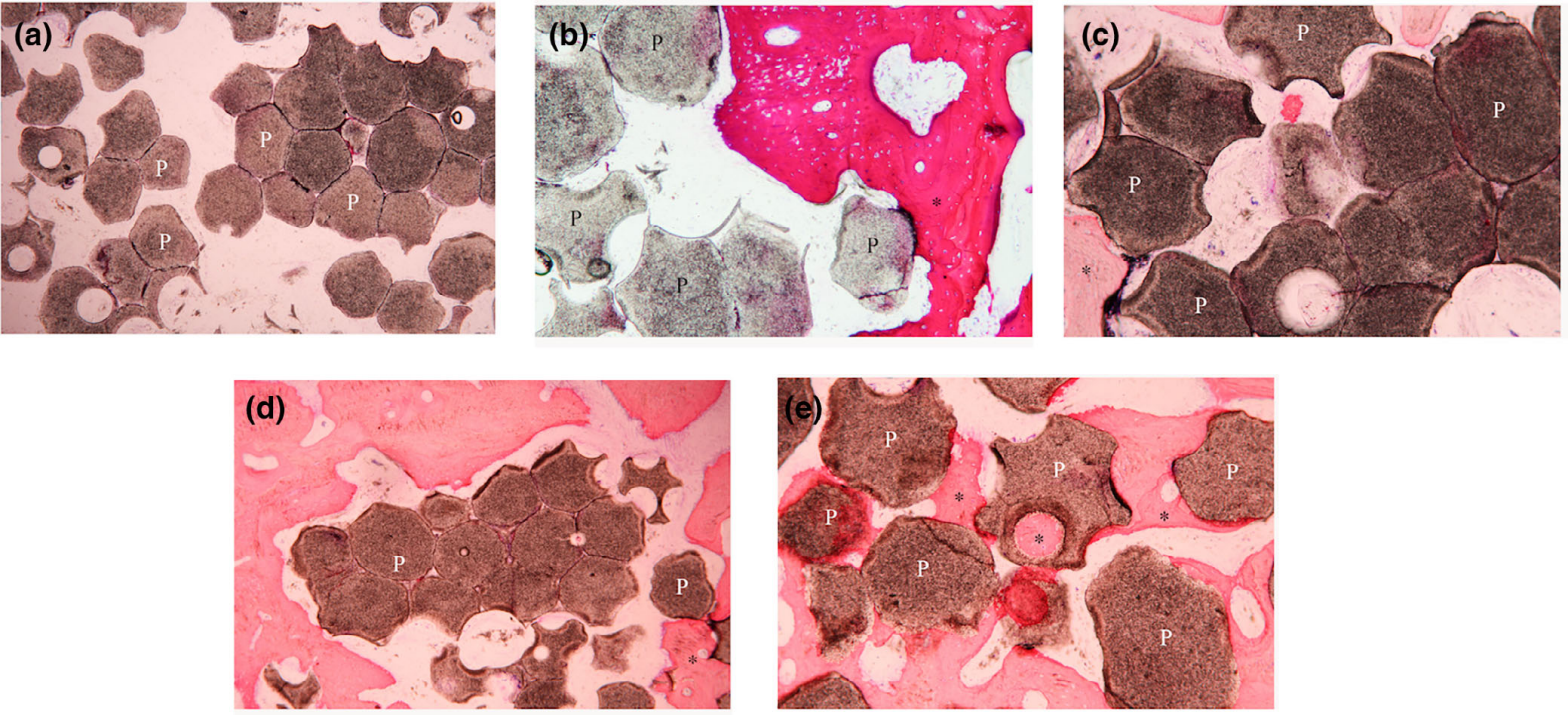
Histologic images show new bone formation (asterisks) and material remnants (P) of HA70 group in (a) 4 weeks. (b) 8 weeks. (c) 12 weeks. (d) 16 weeks: Aggregated particle. (e) 16 weeks: Well‐distributed particle with ×10 magnification

In the fabricated BCP groups, the particles appeared aggregated. In group HA30, new bone was scarcely regenerated during the first 4 week which was located just around the osteotomy site (Figure 6a). The osteoid formation was observed with limited contact to the graft in 8 weeks (Figure 6b). The bone maturation and bone‐to‐graft contact increased over the time (Figure 6b–d). While in group HA70, the woven bone was seen in 16 weeks which showed more quantity in the smaller particles. This group showed restricted contact between bone and graft particles (Figure 7a–e).

**FIGURE 8:**
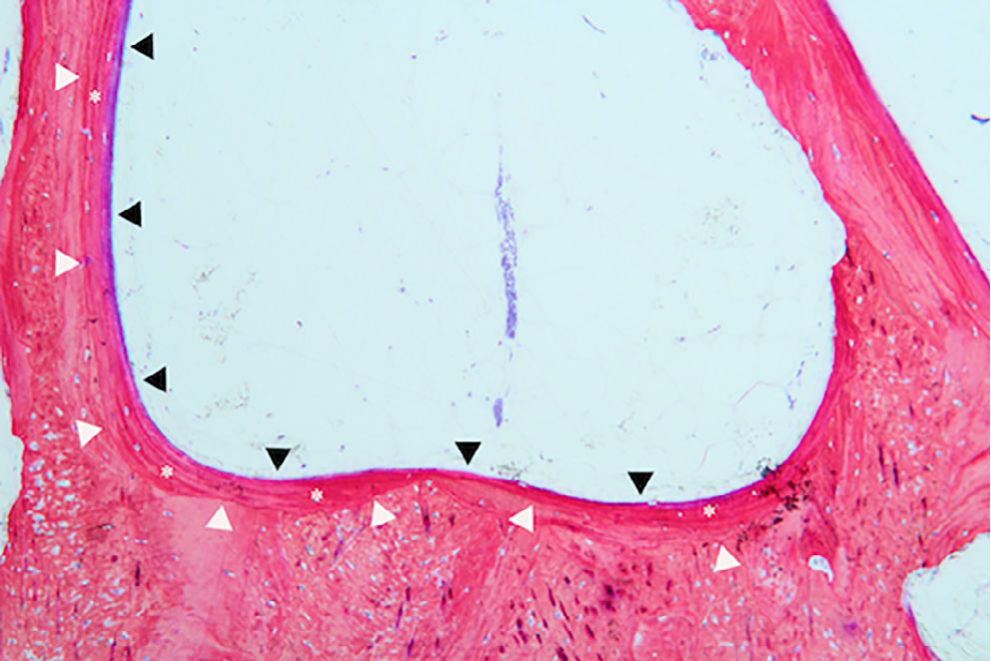
Histologic image of negative group shows soft tissue formation (black triangle), new bone formation (asterisks) and defect outline (white triangle) in 8 weeks with ×10 magnification

In the negative group, the new bone regeneration was very limited. Fibrous tissue growth was mainly observed (Figure 8).

**FIGURE 9:**
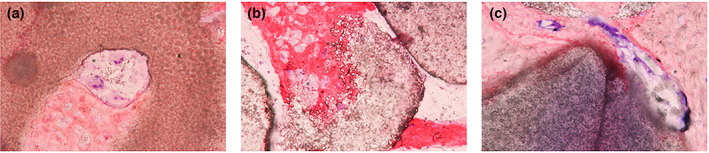
Histologic findings of bone cell activities (the asterisks show the resorption patterns among the bone grafts) in (a) (MBCP group in 12 weeks). (b) (HA30 group in 8 weeks). (c) (HA70 group in 16 weeks) with 40× magnification

All biomaterials have shown good biocompatibility with no adverse fibrous tissue reaction. Furthermore, the bone remodeling process with osteoclastic and osteoblastic reactivity was observed (Figure 9).

The histomorphometric analysis was evaluated. The average measures' value of intraclass correlation was 0.878. The measured values of new bone formation and residual material particles are shown in Table 1, while the percentages of bone‐to‐graft contact are shown in Table 2. The percentage of new bone formation gradually increased with time in the same way with the percentage of bone‐to‐graft contact. Both Kruskal–Wallis and Friedman test found significant differences (*p* < 0.05). Within each time frame, the percentages of new bone formation and residual material particles were not significantly different (Bonferroni correction <0.005). In 16 weeks, the autogenous group trended to show the highest median percentage of new bone formation (45.26 [6.58]), followed by group MBCP (33.52 [2.24]), group HA30 (24.34 [8.04]), group HA70 (19.43 [7.16]), and the negative group (3.37 [0.17]). On the contrary, the median percentage of residual material particles had the highest value in group HA70 (37.03 [2.83]), followed by group HA30 (26.74 [9.03]), group MBCP (17.58 [1.88]) and the autogenous group (1.88 [0.27]). In every time frame, the median percentages of bone‐to‐graft contact was higher in group MBCP, while there were no significant difference found (Bonferroni correction >0.017). In 16 weeks, group MBCP (73.47 [1.77]) showed higher median percentages of bone‐to‐graft contact than group HA30 (41.08 [3.08]) and group HA 70 (14.32[2.12]) with Bonferroni correction <0.017.

**TABLE 1:** Percentage of new bone formation area and percentage of residual material particles (median [interquartile range]) *n* = 8

Time	Histomorphometric analysis	Autogenous	MBCP	HA30	HA70	Negative
4 weeks	% New bone formation	34.80 (13.95)	11.69 (3.52)	6.38 (1.93)	4.13 (0.49)	1.99 (0.07)
	% Residual material particles	12.88 (15.06)	30.80 (4.71)	42.63 (14.76)	43.03 (14.66)	–
8 weeks	% New bone formation	36.53 (33.64)	21.95 (4.48)	18.12 (6.65)	4.41 (0.51)	2.35 (0.11)
	% Residual material particles	3.27 (3.01)	25.45 (2.38)	37.08 (9.61)	41.72 (3.20)	–
12 weeks	% new bone formation	43.16 (13.19)	32.68 (5.68)	21.50 (3.53)	10.80 (3.59)	2.57 (0.60)
	% Residual material particles	1.90 (0.31)	21.14 (5.41)	29.18 (11.10)	40.91 (13.95)	
16 weeks	% New bone formation	45.26 (6.58)	33.52 (2.24)	24.34 (8.04)	19.43 (7.16)	3.37 (0.17)
	% Residual material particles	1.88 (0.27)	17.58 (5.03)	26.74 (9.03)	37.03 (2.83)	–

*Note*: In each time frame, there were no differences between the groups at Bonferroni correction <0.005.

**TABLE 2:** Percentage of bone‐to‐graft contact among biomaterials (median [interquartile range])

Time	MBCP	HA30	HA70
4 weeks	61.31 (3.05)* ^,^ **	11.15 (3.05)*	6.23 (1.21)**
8 weeks	67.12 (1.16)* ^,^ **	15. 34 (1.78)*	7.83 (1.84)**
12 weeks	71.67 (1.20)* ^,^ **	34.86 (1.56)*	9.07 (0.62)**
16 weeks	73.47 (1.77)* ^,^ **	41.08 (3.08)*	14.50 (2.12)**

*Note*: In each time frame, the differences between the groups were analyzed using Mann–Whitney test at Bonferroni correction <0.017.

* Group MBCP was significant different from group HA30 at Asymp. Sig. (two‐tailed) = 0.07.

** Group MBCP was significant different from group HA70 at Asymp. Sig. (two‐tailed) = 0.07.

## 4 DISCUSSION

From XRD patterns, the sharpening to the peak high was associated with high sintering temperatures, while the broadening of the peak width was inversely related to the size of the crystal. Sintering at high temperatures increased the size of the bone graft. As a consequence, the higher peak height and lower peak width value of the fabricated BCP indicates the higher sintering temperature (Mate Sanchez de Val, Calvo‐Guirado, Gomez‐Moreno, Perez‐Albacete Martinez, et al., 2016). This is confirmed by SEM analysis. The two ratios of fabricated BCP show homogenous and high crystalline density which may impair the degradation of the bone graft (Araujo et al., 2010; Dorozhkin, 2007, 2010; Yamada & Egusa, 2017). The SEM images also demonstrated even distribution of macro‐ and microporosity. According to the literature, high porosity enhances cell attachment, cell seeding and cell migration which promotes new bone ingrowth. Moreover, this facilitates the bone graft resorption but too much porosity reduces the mechanical properties (Dorozhkin, 2007, 2010; Hannink & Arts, 2011; Mate Sanchez de Val, Calvo‐Guirado, Gomez‐Moreno, Perez‐Albacete Martinez, et al., 2016). Different pore sizes also play different roles (Klein et al., 2009). Macroporosity is considered essential for bone cell colonization and new bone enhancement (Dorozhkin, 2007; Dorozhkin, 2010; Klein et al., 2009). One study revealed good results in using highly macroporous β‐TCP in maxillary sinus augmentation (Bettach et al., 2014). Microporosity is crucial for protein adsorption (Dorozhkin, 2007, 2010; Klein et al., 2009) and improved growth factor retention (Woodard et al., 2007). An animal study showed significant higher bone formation in microstructured BCP although the remaining graft volume was still higher (Dahlin et al., 2015). Interconnection pores facilitate nutrient transportation, oxygen flow and vasculature (Dorozhkin, 2007, 2010; Klein et al., 2009). A clinical study in maxillary sinus augmentation using interconnective porosity BCP represented new bone ingrowth with vessels within pores (Iezzi et al., 2012). MBCP+TM exhibits nano‐scale porosity in SEM images but these could not be seen in the fabricated BCP. A review suggested that the nanoporous structure could improve cell adhesion, cell proliferation and cell differentiation (Mate Sanchez de Val, Calvo‐Guirado, Gomez‐Moreno, Gehrke, et al., 2016).

Although the well distributed macro‐ and microporosity is fabricated by polymeric sponge method, the median percentage of new bone formation was inferior to MBCP+TM. Pore size and porosity were not the only main focus factors. There were other osteoconductive factors that need to be introduced. The crystallinity from high sintering temperature is another factor as described previously. Grain size and morphology also had an effect on the material resorption (Park et al., 2010). The smaller grain size undergoes faster resorption of the graft which could induce further secondary porosity and promote more new bone regeneration (Mate Sanchez de Val, Calvo‐Guirado, Gomez‐Moreno, Perez‐Albacete Martinez, et al., 2016; Yamada & Egusa, 2017). SEM images show smaller grain sizes in MBCP+TM (500–1000 μm) compared to the fabricated BCP (1000–2000 μm).

In this study, each defect dimension was 5.0 mm in diameter and 2.0 mm in depth following ISO 10993‐6: 2007 protocol to exclude the healing capacity of the bone substitution from the osteotomy site. The sample size was limited due to the animal rights concerns. However, we were able to gather sufficient information within this limitation. The autogenous bone graft trended to show the highest median percentage of new bone formation and exhibited the fastest resorption. This could imply that the autogenous bone graft is still the gold standard for bone regeneration (Cecilia et al., 2018; Corbella et al., 2016; De Risi et al., 2015; Nawas & Schiegnitz, 2014; Papageorgiou et al., 2016; Starch‐Jensen et al., 2018). The median percentage of new bone formation gradually increased among the group. In each time frame, group MBCP, HA30 and HA70 showed no different in values of new bone formation and residual material particles. From histology analysis, the MBCP group performed better distribution in the defects. The aggregation of the materials limits the space for regenerating new bone (Marx, 2007; Roden Jr., 2010). In 4 weeks of the HA30 group, new bone was scarcely regenerated just around the osteotomy site. Since the blood supply comes from this area, the healing potency increased at the osteotomy site (Araujo et al., 2015; Chappuis et al., 2017). In 16 weeks, the woven bone in the HA70 group had a higher quantity of small‐sized particles. The smaller particle size is associated with faster graft resorption and new bone formation (Mate Sanchez de Val, Calvo‐Guirado, Gomez‐Moreno, Perez‐Albacete Martinez, et al., 2016; Yamada & Egusa, 2017). Moreover, the HA70 group trends to degrade with a slower rate than the MBCP and HA30 group, which is a result from the difference among the scaffold material that influence the osteoconductive properties as mentioned earlier. The fabricated BCP was restricted to resorption while MBCP+TM showed better degradation which was consistent with the gradually smaller particle size from histology investigation. This may impair bone regeneration because graft resorption has to take place first. However, this is the advantage for space‐making capacity in long term bone healing (Dorozhkin, 2010; Roden Jr., 2010).

The other significant factor that needs to be mentioned is the composition between HA and β‐TCP. Many studies conclude that lower HA/β‐TCP ratio shows more new bone formation area due to the great dissolubility of β‐TCP (Jelusic et al., 2017; Jensen et al., 2007; Wang et al., 2013; Yang et al., 2014; Zhang et al., 2005; Zhu et al., 2010). In correspondence with this study, the MBCP group composes of the lowest HA/β‐TCP ratio and has a tendency to enhance a higher amount of new bone formation than HA30 and HA70 group which are formulated with a higher ratio of HA respectively. BCP is considered the alternative material. The histology analysis shows good biocompatibility and bone remodeling process among each BCP group. Literature reviews stated that the results in terms of new bone formation and implant survival rate using BCP as a scaffold is comparable to other bone substitutions (Papageorgiou et al., 2016; Starch‐Jensen et al., 2018; Wu et al., 2016). Most clinical studies focus on using BCP in the space‐making defects like maxillary sinus augmentation (Bettach et al., 2014; Chiu et al., 2018; Christer et al., 2009, 2012; Covani et al., 2011; Danesh‐Sani et al., 2016; Frenken et al., 2010; Helder et al., 2018; Iezzi et al., 2012; Lee et al., 2018; Mangano et al., 2013; Nishimura et al., 2018; Ohe et al., 2016; Okada et al., 2017; Schmitt et al., 2013; Sebastian et al., 2010; Sverzut et al., 2015; Tosta et al., 2013).

From a systematic review, BCP gains lower bone‐to‐graft contact than deproteinized bovine bone mineral (DBBM; Wu et al., 2016). However, one study demonstrated a non‐significant difference value (Christer et al., 2012) whereas another study revealed that BCP offers more interfacial bone contact than in DBBM (Yip et al., 2015). No study that evaluated the bone‐to‐graft contact among the BCP was found. This study shows that the median percentage of bone‐to‐graft contact increases overtime among each material. The MBCP group gained more interfacial bone contact than the fabricated BCP with ratio of HA30 and HA70 group respectively. This conforms to the new bone formation area and could be explained by the rationale described earlier.

Clinically, the using of biphasic alloplastic graft is difference in each situation, if the purpose of the use is to maintain the space of the recipient site, the higher percentage of hydroxy apatite is required. In contrary, if the purpose of using alloplastic graft is for small defects and need the faster biomaterial resorption, the higher percentage of tricalcium phosphate is prerfer.

## 5 CONCLUSION

Within the limitation of this study, it can be indicated that the different manufacturing methods result in different results of bone‐to‐graft contact. The polymeric sponge technique can offer well distributed porosity within the graft in terms of macroporosity and microporosity. However, that desired porosity did not correlate with better bone regeneration, the other factors which are crystallinity, grain size and nano‐scale porosity also influence the osteoconductive property.

The autogenous bone graft which remains as the gold standard trends to show the highest new bone formation, while BCP shows good biocompatibility and can be the alternative choice. BCP with higher ratio of β‐TCP has a tendency to result in faster resorption and more new bone formation. However, more sample sizes and long term bone healing should be observed.

**Abbreviations**
BCP: biphasic calcium phosphate
HA: hydroxyapatite
XRD: X‐rays diffraction
β‐TCP: β‐tricalcium phosphate

## CONFLICT OF INTEREST

All authors declare that there are no conflict of interest in this study.

## AUTHOR CONTRIBUTIONS

Punyada Intapibool and Pathawee Khongkhunthian have made substantial contributions to management, analysis and interpretation of data and in collaborating the manuscript. Pathawee Khongkhunthian and Kriangkrai Thongkorn have been participated in the study design and have revised it critically for important intellectual content and also have given final approval of the version to be published. All authors read and approved the final manuscript.

## Data Availability

The data that support the findings of this study are available from the corresponding author upon reasonable request.
